# Impact of Toll-Like Receptor-Specific Agonists on the Host Immune Response to the *Yersinia pestis* Plague rF1V Vaccine

**DOI:** 10.3389/fimmu.2021.726416

**Published:** 2021-08-27

**Authors:** Sergei Biryukov, Jennifer L. Dankmeyer, Zain Shamsuddin, Ivan Velez, Nathaniel O. Rill, Raysa Rosario-Acevedo, Christopher P. Klimko, Jennifer L. Shoe, Melissa Hunter, Michael D. Ward, Lisa H. Cazares, David P. Fetterer, Joel A. Bozue, Patricia L. Worsham, Christopher K. Cote, Kei Amemiya

**Affiliations:** ^1^Bacteriology Division, United States Army Medical Research Institute of Infectious Diseases, Frederick, MD, United States; ^2^Molecular Biology Division, United States Army Medical Research Institute of Infectious Diseases, Frederick, MD, United States; ^3^Biostatistics Division, United States Army Medical Research Institute of Infectious Diseases, Frederick, MD, United States

**Keywords:** *Yersinia pestis*, plague, mice, F1-V antigen, vaccine, CpG, immune response, toll-like receptors

## Abstract

Relatively recent advances in plague vaccinology have produced the recombinant fusion protein F1-V plague vaccine. This vaccine has been shown to readily protect mice from both bubonic and pneumonic plague. The protection afforded by this vaccine is solely based upon the immune response elicited by the F1 or V epitopes expressed on the F1-V fusion protein. Accordingly, questions remain surrounding its efficacy against infection with non-encapsulated (F1-negative) strains. In an attempt to further optimize the F1-V elicited immune response and address efficacy concerns, we examined the inclusion of multiple toll-like receptor agonists into vaccine regimens. We examined the resulting immune responses and also any protection afforded to mice that were exposed to aerosolized *Yersinia pestis*. Our data demonstrate that it is possible to further augment the F1-V vaccine strategy in order to optimize and augment vaccine efficacy.

## Introduction

Plague is an acute, febrile, and contagious disease caused by the gram negative bacterium, *Yersinia pestis*, whose natural life cycle includes rodents and fleas (1, 2). Plague is endemic in various parts of the world including Africa, North and South America, and Asia (3). Humans often become infected after a bite from a *Y. pestis*-infected flea, but also through the consumption of uncooked contaminated meat or contact with the infectious animal (2). Depending on the route of infection, plague can manifest as three distinct clinical forms in humans: bubonic, septicemic and pneumonic, the latter of which being the most lethal form of the disease (3). The aerosols produced by a pneumonic plague patient contain large numbers of *Y. pestis* bacilli, which can infect other people (4). The administration of effective antibiotics and preventive therapies are crucial within 24 hours of onset of symptoms (2). In the absence of appropriate antibiotic treatment, pneumonic plague mortality approaches 100% (5). Because *Y. pestis* is highly infectious by the inhalational route, it is a recognized public-health concern and considered a potential biological threat agent for both the military and civilian populations (3).

There was one licensed vaccine available in the US, which was prepared from formaldehyde killed organisms preserved in phenol (6). Due to questionable efficacy and considerable reactogenicity, this vaccine is no longer used. Accordingly, there are several vaccines under development (7–9). The former Soviet Union, along with other nations have traditionally focused on live attenuated vaccines, with tens of millions of individuals receiving the live attenuated plague vaccine, the NIIEG line of the *pgm*‐negative strain EV76. Live attenuated vaccines are able to confer protection against bubonic and pneumonic infection, however the protection appears to be short lived and highly reactogenic in some vaccinees (10, 11).

Vaccination with subunit vaccine candidates is considered to be an effective strategy for long term protection (7). Currently, the recombinant plague subunit vaccine development is largely centered on two antigens: F1 and V (12, 13). F1, the capsular antigen of *Y. pestis*, appears to prevent phagocytosis of plague bacilli. The V antigen forms the tip of the *Y. pestis* type three secretion system (T3SS) and has a key role in the translocation of the cytotoxic Yersinia outer proteins (Yops) into host cells, as well as stimulating the production of immunosuppressive cytokines (14–16). These antigens have been studied alone, as well as both a co-mixture (F1+V), and as a fusion protein (F1-V or rF1V) (17, 18). The rF1V vaccine is formulated with Alhydrogel, an adjuvant approved by the United States Food and Drug Administration (FDA) and used in most of the plague vaccine studies reported to date (19). Animal studies and Phase I human clinical trials found subunit vaccine strategies including F1 and V to be safe (20–23). In some cases, an altered form of V lacking the purported immunomodulating function has been used in vaccine studies and appears to retain antigenicity (24, 25). Vaccination with the rF1V fusion protein induced a strong antibody response but a poor cell mediated immune (CMI) response, nonetheless rF1V protected mice and some non-human primate species against bubonic and pneumonic plague with a F1+ *Y. pestis* CO92 strain (26–29). Additionally, in a trial against aerosolized F1+ (encapsulated) or F1- (non-encapsulated) strains of *Y. pestis*, rF1V vaccination failed to consistently protect African green monkeys, whereas cynomolgus macaques were more readily protected against the F1+ strain CO92 (30). These results generated concerns about the inadequacy and inconsistency of protection afforded by the rF1V-based vaccine. Additional concerns have been raised regarding the protection this vaccine would provide against exposure to genetically engineered or naturally occurring F1-negative strains as these F1-negative strains retain virulence despite the loss of capsule (31–33). Likewise, with an rF1V-based vaccine protection against F1-negative strains relies entirely on the V antigen component of the fusion protein. Data available in the literature do not allow us to draw substantiated conclusions regarding the efficacy of rF1V vaccine against challenge with non-encapsulated strains. There were similar concerns with the previously licensed formaldehyde-killed whole cell plague vaccine, USP, where the protective antigen in the preparation was shown to be primarily the F1 capsular antigen and the vaccine in some cases was poorly protective against pneumonic plague (34–36). Due to the questionable efficacy of the rF1V in nonhuman primates and against different strains of *Y. pestis*, a more efficacious plague vaccine is needed that can induce both an enhanced antibody response and cell-mediated immunity in large animal models of pneumonic plague. We hypothesize that this could be achieved by incorporating immunomodulators in rF1V vaccine formulations to promote the induction of a more robust immune response.

Adjuvants (e.g. alhydrogel) are compounds that enhance immunization to vaccines and experimental antigens for specific pathogen by a variety of mechanisms (37). The resulting immune cascade identifies pathogen-associated molecular patterns (PAMPs) through pathogen-recognition receptors (PRRs), including the Toll-like receptors (TLRs). Because of their role in pathogen recognition and activation of the innate and adaptive immune response, TLR agonists are utilized to augment immunization as vaccine adjuvants.

Klinman et al., demonstrated that CpG oligodeoxynucleotides (ODNs), can augment the immune response to Alhydrogel-formulated vaccines (38). ODNs are TLR9-dependent immunomodulators that have been proven to enhance the host’s ability to resist infection by accelerating and improving the induction of an innate and adaptive immune responses, characterized by the production of T helper cell (Th1)-promoting pro-inflammatory cytokines. In a bubonic plague model, CpG ODNs included in a prime-boost vaccine strategy significantly augmented the immune response leading to protection of rF1V vaccinated mice against subcutaneous *Y. pestis* CO92 challenge and shifted the immune response towards a more protective Th1-like polarization (19).

In this study, we compared the level of protection against *Y. pestis* CO92 *versus Y. pestis* C12 (F1-negative, non-encapsulated strain of *Y. pestis*) aerosol challenge in mice vaccinated with rF1V. Secondly, we assessed a select number of TLR agonists as adjuvants with the rF1V subunit vaccine by monitoring their ability to enhance both the antibody and cellular immune responses, and the level of protection against *Y. pestis* C12 aerosol challenge. We predicted that the addition of a TLR agonist will augment antibody-mediated response against the V antigen, thereby enhancing protection against the F1-negative *Y. pestis* C12 strain.

## Methods

### Bacterial Strains and Media

*Y. pestis* CO92 (39) and its non-encapsulated derivative strain C12 (32) were used in this study. Broth cultures were inoculated using growth from freshly inoculated tryptose blood agar base slants which were suspended in Heart Infusion broth + 0.2% Xylose (HIBX) and incubated approximately 24h at 28-30°C and shaking at 150 rpm.

### Animals and Vaccinations

Female, 6–8-week-old BALB/c and CD-1 mice were purchased from the Charles River Laboratories (Frederick, MD). Mice were vaccinated once or twice (three weeks apart), depending on the study, subcutaneously (s.c.) with rF1-V, in the presence or absence of TLR Agonists **(** **Table S1** **)**, and 250 - 500 µg of Alhydrogel (Brenntag Biosector, Denmark) in a total volume of 0.1 ml. PolyICLC (Hiltonol™) was provided by Oncovir, Inc., whereas Pam3CSK4, MPLA, CpG2006, Imiquimod and R848 were purchased from Invivogen and reconstituted per manufacturer’s recommendation. The number of mice, the amount of rF1-V, and TLR Agonists for each study are stated in the figure legends or tables. After 25 to 28 days post vaccination, depending on the study, blood was obtained by intracardiac or axillary vessel collection for serum isolation and antibody titer determination from deeply anesthetized mice. In addition, spleens were harvested for cytokine profiling from select studies. After 26-43 days, depending on the study, mice were challenged *via* s.c. or aerosol routes. The calculated colony-forming units (CFU) doses for each study are stated in the figure legends.

### Exposure of Mice to Virulent *Y. pestis* Challenge

Aerosolized challenge doses of virulent *Y. pestis* (pneumonic plague model) were prepared as previously described (40, 41). The cultures were harvested by centrifugation and suspended in HIB medium (no xylose) to the concentration yielding the number of LD_50_ doses indicated in the tables. Exposure of mice to aerosolized bacteria was accomplished as previously described (40–42). Briefly, mice were transferred to wire mesh cages and placed in a whole-body aerosol chamber within a class three biological safety cabinet located inside a BSL-3 laboratory. Mice were exposed to aerosols of either *Y. pestis* strain CO92 (encapsulated) or C12 (non-encapsulated) created by a three-jet collision nebulizer. Up to 48 mice can be exposed at one time to aerosolized *Y. pestis*. Samples were collected from the all-glass impinger (AGI) vessel and analyzed by performing CFU calculations to determine the inhaled dose of *Y. pestis.* The inhaled dose was calculated using Guyton’s formula: Respiratory volume per minute in ml = 2.10 x (weight in grams)^0.75^ (43, 44). The estimated inhaled doses are listed in each experimental iteration and represent the average delivered dose to the mice in that experimental iteration.

Cohorts of mice were also challenged *via* the subcutaneous route (bubonic plague model). These mice were inoculated with 0.2 ml volumes of potassium phosphate buffer (Kphos) that contained CFU harvested directly from the tryptose agar blood base slants with no further culturing (40, 41). All mice, regardless of route of exposure to *Y. pestis*, were observed for 21 days post exposure to virulent *Y. pestis* and early endpoint euthanasia was performed in accordance with approved euthanasia criteria.

Research was conducted in compliance with the Animal Welfare Act and other federal statutes and regulations relating to animals and experiments involving animals and adheres to principles stated in the *Guide for the Care and Use of Laboratory Animals*, National Research Council, 2011. The facility where this research was conducted is fully accredited by the Association for Assessment and Accreditation of Laboratory Animal Care International.

### Spleen Cell Preparation

Splenocytes were prepared based on a previously published protocol (45). Briefly, spleens were excised from mice (n=5 mice per group), weighed, and disaggregated in RPMI 1640 medium (ThermoFisher, Grand Island, NY). Red cells in the spleen homogenate were lysed with Ammonium-Chloride-Potassium (ACK) Lysing Buffer (BioWhittaker, Walkersville, MD) after the extract was diluted with RPMI 1640 medium and cells pelleted by centrifugation at 335 xg (1,200 rpm) for 10 min. Splenocytes were then re-suspended in complete medium RPMI (1640 medium containing 10% heat-inactivated fetal calf serum (ThermoFisher), 1 mM sodium pyruvate, 0.1 mM non-essential amino acids, 100 U/ml of penicillin/100 µg/ml streptomycin, and 50 µM 2-mercaptoethanol and the cells counted.

### Cytokine Analyses: Luminex

Interferon gamma (IFN-γ) expression profiles were measured by the Luminex MagPix (ThermoFisher, Grand Island, NY). Briefly, splenocytes were plated in 48 well plates (Corning, FisherSci) at 10^6^ cells/well and were stimulated with rF1V (25 μg/mL) in RPMI complete media for 48 hrs. The cells were centrifuged at 1200 xg for 10 min, the supernatant was collected, and then processed as per the IFN-γ Mouse ProcartaPlex Simplex Kit protocol (ThermoFisher). The concentration of the cytokines in the supernatant samples was calculated by plotting the expected concentrations of the standards against the MFI generated by each standard. A five-parameter logistic (5PL) standard curve was used for best curve fit model.

### Cytokine Analyses: ELiSpot

Interferon gamma (IFN-γ) expression was measured by seeding purified splenocytes in the presence of rF1V. Briefly, 96-well plates were coated overnight at 4°C with 80 µl/well capture anti-mouse IFN-γ monoclonal antibody. Plates were washed one time with 1x PBS. rF1V (25 µg/ml) was re-suspended in CTL-Medium with 1% L-Glutamine and 100 µl was added to each well. The plates were incubated at 37°C, 9% CO_2_ for 15 min. Splenocytes were resuspended in CTL-Medium with 1% L-Glutamine and seeded at 8x10^5^ cells per well for rF1V stimulation. Plates were incubated for 24 hr at 37°C 9% CO_2_ and splenocytes were removed and plates were washed twice with PBS alone and then twice with PBS and 0.05 % Tween. 80 μL/well biotinylated detection anti-mouse IFN-γ monoclonal antibody was added. After 2 hr incubation at room temperature (RT), plates were washed three times with PBS and 0.05 % Tween. 80 μl of Strep-AP antibody solution was added to the wells, and the plates were incubated for 30 min at RT. Development reagents were added and incubated for 15 min at RT according to manufacturer recommendations. The colorimetric reaction was stopped by washing the plates three times with distilled water and air drying overnight. Spots were scanned and analyzed using an automated ELISPOT reader (CTL-Immunospot S6 Analyzer, CTL, Germany). The splenocyte response was assessed as spot forming cells (SFC), adjusted to 10^6^ cells per well, which was automatically calculated by the ImmunoSpot^®^ Software for each stimulation condition and the medium only control.

### ELISA

Immunoglobulin (Ig) class IgG titers (IgG, IgG1, IgG2a) from vaccinated mice were determined by an ELISA performed in 96-well, Immulon 2 HB, round-bottom plates (ThermoFisher). rF1V (cGMP; DynPort Vaccine Company, Frederick, MD), F1 (BEI Resources, Manassas, VA), and V (BEI Resources, Manassas, VA) were individually used as antigens diluted in 0.1 M carbonate buffer, pH 9.5, to a concentration of 2 µg/ml. Plates were covered and stored overnight at 4° C. The plates were washed five times with 1X wash buffer (1× PBS, 0.05% Tween 20) with a Biotek ELx405ts plate washer (Winooski, VT), and incubated with blocking buffer (1% Casein in PBS, Pierce/FisherScientific) for 30 min at 37°C. Blocking buffer was removed by washing as stated above, then twofold serial dilutions of mouse sera were made with antibody assay diluent (1X PBS, 0.25% Casein) in triplicate, and plates were incubated for 1 h at 37°C. Then the plates were washed as previously mentioned; diluted anti-IgG, -IgG1, or -IgG2a horseradish peroxidase conjugate (1:5,000; Southern Biotechnology Associates, Inc. Birmingham, AL) was added to each well and plates were incubated for 30 min at 37°C. After the plates were washed as previously stated, buffered hydrogen peroxide and 3,3′,5,5′-tetramethylbenzidine solution (Pierce, ThermoFisher) was added to each well, and plates were incubated for 20 min at 37°C. The reaction was stopped with 2 N sulfuric acid, and the amount of bound antibody was determined colorimetrically by reading at 450 nm with a reference filter (570 nm) with a Biotek ELx808 plate reader (Winooski, VT). The results are reported as the reciprocal of the highest dilution giving a mean OD of at least 0.1 (which was at least twice the background) ± 1 SD.

### Mass Spectrometry Sample Preparation

rF1V dilutions of between 5 and 100 µg protein in either 0 or 100 µg Alhydrogel were processed by Filter-Aided Sample Preparation (FASP) (46). Briefly, reaction supernatant (50 µL) was reduced with 5 mM DTT @ 55°C and cysteine alkylated with 25 mM iodoacetamide and added to 200 µL 8 M Urea/100 mM Tris–HCL pH 8.5 (Solution UT8) and filtered through a Microcon-30 kDa Centrifugal Filter Unit with an Ultracel-30 membrane (Millipore, MRCF0R030) at 14,000 × G for 15 min. Following several washing steps with 100 mM Tris pH 8.0, samples were digested with 0.5 µg Trypsin/Lys-C (Promega, V5071) overnight at 37°C. Samples were purified by C18 spin column, dried to completion by speed-vac and stored at − 20 °C until analyzed by LC MS/MS.

### LC–MS/MS Analysis

Sample digests were re-suspended in 40 μL of 0.1% formic acid in HPLC grade water (Buffer ‘A’). A Dionex 3000 RSLCnano system (Thermo Scientific) injected 5 μL of each digest onto a pre-column (C18 PepMap 100, 5 μm particle size, 5 mm length × 0.3 mm internal diameter) using a flow rate of 10 µL/min. Peptides were then loaded onto an Easy-Spray analytical column (15 cm × 75 um) packed with PepMap C18, 3 um particle size, 100 A porosity particles (Thermo Scientific, Inc.). A 5–60% B gradient elution in 60 min was formed using mobile phase A and B (85% acetonitrile in 0.1% formic acid) at a flow rate of 300 nL/min. The column eluent was connected to an Easy-Spray source (Thermo Scientific) with an electrospray ionization voltage of 2.2 kV. An Orbitrap Elite mass spectrometer (Thermo Scientific, Inc.) with an ion transfer tube temperature of 300 °C and an S-lens setting of 55% was used to focus the peptides. A top 20 data dependent MS/MS method was used to select the 20 most abundant ions in a 400–1600 amu survey scan (60,000 resolution FWHM at m/z 400) with a full AGC target value of 10^6^ ions and a maximum injection time of 200 ms. MS/MS spectra were acquired at a resolution of 60,000 (FWHM at m/z 400) with an AGC target value of 5x10^5^ ions and a maximum injection time of 200 ms. The isolation width for MS/MS CID fragmentation was set to 2 Daltons. The normalized Collision energy was 35% with an activation time of 0.1 ms. The dynamic exclusion duration was 30 s.

### Database Search and Protein Quantitation

Acquired MS/MS protein searches were performed with ProteomeDiscoverer 2.1 Sequest HT (Thermo Scientific) using a *Y. pestis* (taxID 632, 3,726 proteins) and Human (taxID 9606, 20,376 proteins) subset of the SwissProt_2017_01_18 database Variable modifications used were Carbamyl (KMR), Methyl (DE), Acetyl (K), Deamidated (NQ), and Oxidation (M). Cysteine carbamidomethylation was specified as a constant modification. The peptide-level false discovery rate (FDR) was set at 0.1% using Posterior Error Probability validation. Only proteins having at least 2 Peptide Spectral Matches (PSM) were considered, with both unique and razor peptides used for spectral counts. Mass tolerances were 10 ppm for the MS1 scan and 0.6 Da for all MS/MS scans. Relative quantitation was performed using spectral peptide match counting of F1 or V peptides using the criteria above.

### Statistical Analysis

IFN-γ values were log-transformed for analysis and were summarized as geometric means (Geo Mean) and geometric standard error (GSE), and p-values indicate the main effect contrasts from a two-way ANOVA (treatment by assay date). ELISA antibody titers were compared by Welch’s *t*-test on log transformed values and were summarized as Geo Mean and GSE. Fisher exact tests and log-rank tests were used to compare mouse survival curves and time to death post challenge, respectively. Median lethal doses (LD_50_) were estimated by probit regressions, with confidence limits by the delta method. Analysis was implemented in SAS version 9.4 (SAS Institute Inc., Cary, NC).

## Results

### Inbred BALB/c Mice Are More Susceptible to *Y. pestis* Challenge Than the Outbred CD1 Mice

To facilitate subsequent studies on vaccine efficacy and future improvements to the formulation, the susceptibility of inbred BALB/c and outbred CD-1 mice to F1+ (CO92) strain of *Y. pestis* was evaluated (47, 48). The median lethal dose (LD_50_) aerosol values of *Y. pestis* CO92 are similar in BALB/c (6.80x10^4^ CFU) and CD-1 mice (3.30x10^4^ CFU) (**Table S2**). A single dose vaccination schedule for BALB/c and CD-1 mice was implemented with the goal of identifying a reasonable rF1V antibody dose response and corresponding susceptibility to *Y. pestis* CO92 challenge. A single subcutaneous dose of rF1V was administered to BALB/c and CD-1 mice and the animals were exposed to aerosolized *Y. pestis* CO92, 26 days later. The groups were as follows: Control (PBS), 0.1 µg rF1V, 1.0 µg rF1V or 10 µg rF1V. All vaccine preparations including the PBS group contained 500 µg/dose of *Alhydrogel.*

**Figure 1:**
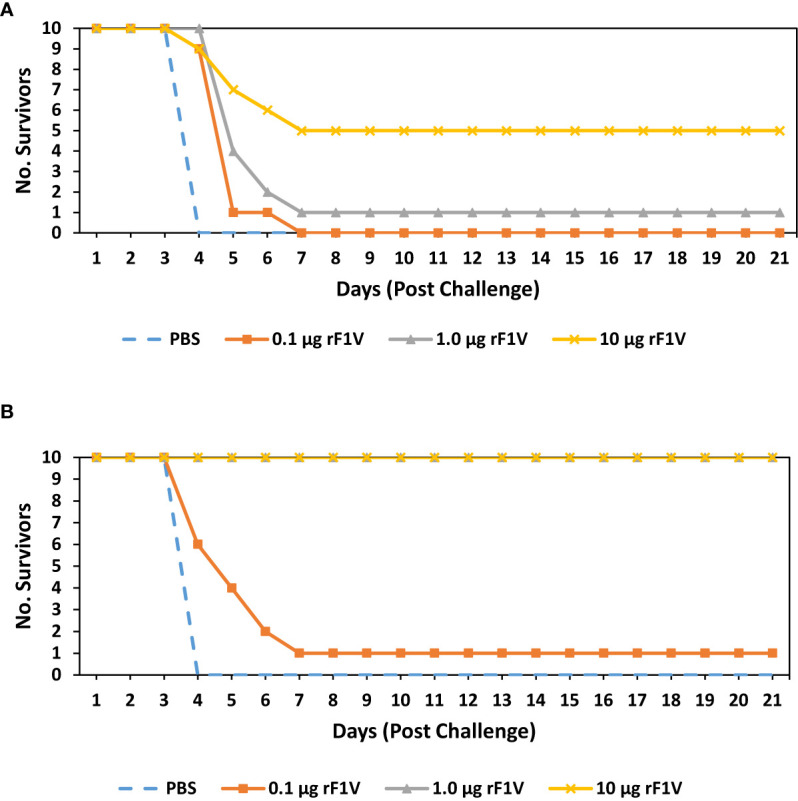
BALB/c mice vaccinated with rF1V are more susceptible than CD1 vaccinated mice to *Y. pestis* infection. **(A)** BALB/c or **(B)** CD1 mice were vaccinated once, subcutaneously, with mock/PBS, 0.1 µg, 1 µg or 10 µg of rF1V (n=10). At 26 days post vaccination, BALB/c and CD1 mice were exposed to 2.02x10^6^ CFU (approximately 30 LD_50_ equivalents) or 1.75x10^6^ CFU (approximately 53 LD_50_ equivalents) of aerosolized *Y. pestis* CO92, respectively. Survival was monitored for 21 days post challenge.

Both BALB/c and CD-1 mice were exposed to aerosolized bacteria 26 days post vaccination (BALB/c mice received approximately 30 LD_50_ [2.02x10^6^ CFU] and CD-1 mice received approximately 53 LD_50_ [1.75x10^6^ CFU]). Mice were monitored for 21 days post challenge. All (10/10) BALB/c control group mice challenged with *Y. pestis* CO92 succumbed or were euthanized within 4 days of infection while all mice vaccinated with 0.1 µg rF1V died or were euthanized within 7 days post challenge (p=0.2 *vs* PBS for). Of the remaining rF1V vaccinated mice that were challenged with *Y. pestis* CO92, 1/10 (p=0.1 *vs* PBS) and 5/10 (p<0.001 *vs* PBS) mice survived the challenge that were vaccinated with 1.0 µg and 10 µg of rF1V, respectively (**Figure 1A**). All (10/10) CD-1 control group mice challenged with *Y. pestis* CO92 succumbed or were euthanized within 4 days of infection. Only 1/10 (p=0.1 *vs* PBS) CD-1 mice vaccinated with 0.1 µg rF1V survived, but despite a higher challenge dose, all (p<0.0001 *vs* PBS) mice in the 1.0 µg and 10 µg rF1V groups survived for the duration of the study **(** **Figure 1B** **)**.

On the day prior to challenge (day 25), 4 mice from each group were euthanized and assayed for antibody titers against rF1V. Antibody response (IgG) increased with increasing vaccine dose in both BALB/c and CD-1 mice. Relative to BALB/c mice, CD-1 mice had a significantly greater overall antibody response at 1.0 µg (9.0x10^4^ BALB/c *vs*. 6.4x10^5^ CD-1; p<0.0001) and 10 µg (1.6x10^5^ BALB/c *vs*. 9.6x10^5^ CD-1; p<0.001) rF1V dose which likely contributed to the higher level of protection against *Y. pestis* CO92 challenge **(** **Table 1** **)**. Furthermore, IgG1 titers were also higher in CD-1 *versus* BALB/c mice but IgG2a titers were lower. Statistical significance in antibody titers was reached for all groups within both strains of mice relative to PBS vaccinated group with the exceptions of antibody titers in CD-1 mice vaccinated with 0.1 µg rF1V dose (p=0.05-0.1) and in IgG2a titers in CD-1 mice vaccinated with 1.0 µg rF1V dose (p=0.1). Overall, the predominant antibody response was IgG1 biased in both strains of mice. Due to the high level of protection seen in CD-1 mice, the more susceptible BALB/c mouse model was chosen for further studies.

**Table 1:** Antibody titers after a single vaccination with rF1V before aerosol challenge.

Treatment^a^ ^,^ ^b^	Mouse Strain	IgG^c^		IgG1^c^		IgG2a^c^		Ratio IgG2a/IgG1
		Geo Mean (GSE)	*vs*. PBS (p-Value)	Geo Mean (GSE)	*vs*. PBS (p-Value)	Geo Mean (GSE)	*vs*. PBS (p-Value)	
PBS	BALB/c	<50	N/A	53 (1.06)	N/A	<50	N/A	NA
	CD-1	<50	N/A	59 (1.19)	N/A	<50	N/A	NA
	*vs*. BALB/c (p-Value)		1.0000		0.6219		1.0000	
0.1 μg rF1V	BALB/c	1,270 (1.76)	0.0002	80,476 (1.91)	<.0001	800 (1.51)	0.0002	0.0099
	CD-1	212 (2.33)	0.054	168 (2.04)	0.1252	126 (2.04)	0.1312	0.75^d^
	*vs*. BALB/c (p-Value)		0.0875		<.0001		0.0382	
1.0 μg rF1V	BALB/c	90,510 (1.43)	<.0001	269,087 (1.19)	<.0001	2,525 (3.90)	0.0192	0.0094
	CD-1	640,000 (1.10)	<.0001	800,000 (1.35)	<.0001	844 (5.29)	0.0804	0.001
	*vs*. BALB/c (p-Value)		<.0001		0.0102		0.6178	
10 μg rF1V	BALB/c	163,326 (1.21)	<.0001	955,134 (2.09)	<.0001	7,551 (1.19)	0.0001	0.0079
	CD-1	957,023 (1.51)	<.0001	5,079,683 (1.75)	<.0001	597 (4.35)	0.0287	0.0001
	*vs*. BALB/c (p-Value)		0.0009		0.0799		0.1024	

a n was 4 for each group of mice.

b Single subcutaneous vaccination.

c Titer 26 days after vaccination, IgG from 1 of 2 repeats done in triplicate. Reported as geometric mean (Geo Mean) with geometric standard error (GSE).

d We postulate that the exagerated IgG2a/IgG1 ratio is an artifact due to the low titers.

### BALB/c Mice Vaccinated With rF1V Are Less Protected Against the Non-Encapsulated *Y. pestis* C12 Strain

It is important when developing new vaccines, particularly those designed to protect against biological threat agents, that we address the issue of vaccine resistance or escape brought about by natural selection or by the genetic engineering of strains. One of the concerns with the previously licensed killed whole cell plague vaccine, USP, was that the protective antigen in the preparation was primarily the F1 capsular antigen (12). Although the vaccine was protective against wild-type *Y. pestis* in several animal models, there is no evidence that it could protect against genetically engineered or naturally occurring F1-negative strains to the same level (49, 50). Non-encapsulated strains maintained virulence despite the loss of capsule (51–53). With an rF1V-based vaccine, protection against F1-negative strains relies entirely on the V antigen component of the fusion protein. To this end, we compared the virulence of *Y. pestis* CO92, our wild-type strain, to that of *Y. pestis* C12, which is the capsule negative (F1 deficient) strain, whereby the immune response generated by rF1V would be exclusively reliant on the V portion of the vaccine to protect against *Y. pestis* C12 challenge. We determined LD_50_ values for the C12 strain in both BALB/c mice and CD-1 mice by both subcutaneous (bubonic) and aerosol (pneumonic) routes (**Table S2**).

**Figure 2:**
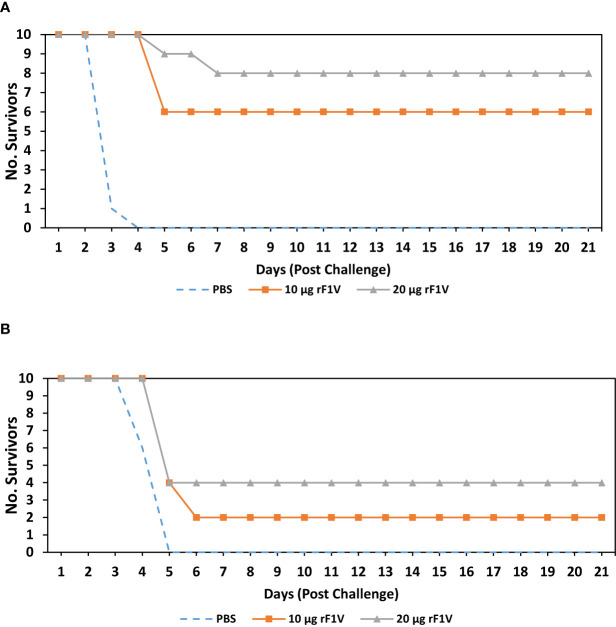
BALB/c mice vaccinated with rF1V are more susceptible to non-encapsulated *Y. pestis* C12 strain. BALB/c mice were vaccinated once, subcutaneously, with mock/PBS, 10 µg or 20 µg of rF1V (n=10). At 29 days post vaccination, BALB/c mice were exposed to aerosolized bacteria with **(A)** 2.63x10^6^ CFU of *Y. pestis* CO92 (approximately 39 LD_50_ equivalents) or **(B)** 2.31x10^6^ CFU of *Y. pestis* C12 (approximately 30 LD_50_ equivalents), respectively. Survival was monitored for 21 days post challenge.

We vaccinated BALB/c mice with a single subcutaneous dose of rF1V (10 µg rF1V, or 20 µg rF1V). All vaccine preparations including the PBS group contained 500 µg/dose of *Alhydrogel.* The mice were exposed to aerosolized bacteria 29 days post vaccination with *Y. pestis* CO92 (Inhaled dose = 2.63x10^6^ CFU or approximately 39 LD_50_) or *Y. pestis* C12 (Inhaled dose = 2.31x10^6^ CFU or approximately 30 LD_50_). Mice were monitored for 21 days post challenge. All (10/10) control group mice challenged with *Y. pestis* CO92 succumbed or were euthanized within 4 days of infection. Of the rF1V vaccinated mice that were challenged with *Y. pestis* CO92, 6/10 (p=0.0001 *vs* PBS) and 8/10 (p<0.0001 *vs* PBS) mice survived the challenge that were vaccinated with 10 µg and 20 µg of rF1V, respectively **(** **Figure 2A** **)**. Of the mice challenged with *Y. pestis* C12, all (10/10) control-infected animals succumbed to infection or were euthanized within 5 days of challenge, while 2/10 (p= 0.0208 *vs* PBS) and 4/10 (p=0.0019 *vs* PBS) mice survived the challenge that were vaccinated with 10 µg and 20 µg of rF1V, respectively **(** **Figure 2B** **)**. Based on survival analyses, the capsule negative (F1 deficient) *Y. pestis* C12 strain is more refractory to a single vaccine dose of rF1V than wild-type *Y. pestis* CO92 strain.

### Double Dose of rF1V Enhances Protection Against *Y. pestis* CO92 and C12 Strains

**Figure 3:**
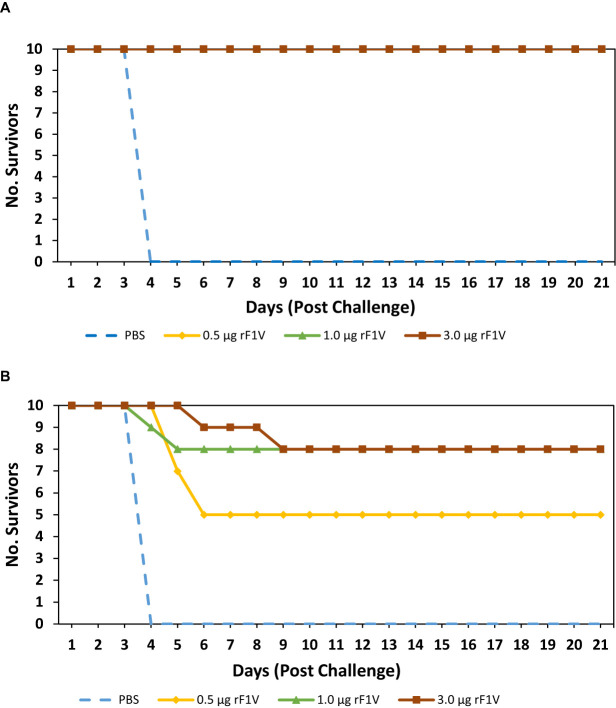
Double dose of rF1V enhances protection against *Y. pestis* challenge. BALB/c mice were vaccinated with two subcutaneous doses, 3 weeks apart with mock/PBS, 0.5 µg, 1.0 µg or 3.0 µg of rF1V (n=10). At 29 days post vaccination, BALB/c mice were exposed to aerosolized bacteria with **(A)** 4.53x10^5^ CFU (approximately 7 LD_50_ equivalents) of *Y. pestis* CO92 or **(B)** 6.09x10^5^ CFU (approximately 8 LD_50_ equivalents) of *Y. pestis* C12, respectively. Survival was monitored for 21 days post challenge.

Our next objective was to determine the level of antibody titers required to achieve 100% protection from an aerosol challenge with *Y. pestis* CO92 and *Y. pestis* C12. To this end, we vaccinated 4 groups of BALB/c mice with two subcutaneous doses of rF1V, 3 weeks apart. The groups were as follows: Control (PBS), 0.5 µg rF1V, 1.0 µg rF1V and 3.0 µg rF1V. All vaccine preparations, including the PBS group contained 500 µg/dose of *Alhydrogel.* The mice were challenged with aerosolized bacteria 29 days post boost vaccination with *Y. pestis* CO92 (Inhaled dose approximately 7 LD_50_s [4.53x10^5^ CFU]) or *Y. pestis* C12 (Inhaled dose = approximately 8 LD_50_s [6.09x10^5^ CFU]). Mice were monitored for 21 days post challenge. All (10/10) control group mice challenged with *Y. pestis* CO92 died or were euthanized within 4 days of infection. Of the rF1V vaccinated mice that were challenged with *Y. pestis* CO92, 10/10 (p<0.0001 *vs* PBS) mice in all groups survived the challenge that were vaccinated with 0.5 µg, 1.0 µg and 3.0 µg of rF1V **(** **Figure 3A** **)**. Of the mice challenged with *Y. pestis* C12, all (10/10) control-vaccinated animals succumbed to infection within 4 days of challenge, while 5/10 (p=0.0017 *vs* PBS) of the 0.5 µg rF1V vaccinated mice and 8/10 (p<0.0001 *vs* PBS) of the 1.0 µg and 3.0 µg of rF1V vaccinated mice survived the challenge **(** **Figure 3B** **)**. These results demonstrate that a double dose regimen of rF1V confers a greater level of protection against both *Y. pestis* CO92 and *Y. pestis* C12 relative to a single dose vaccine regiment. Although, protection is appreciably lower in mice challenged with the F1-negative (capsule deficient) *Y. pestis* C12 strain.

On the day prior to challenge, 4 mice from each group were euthanized and assayed for antibody titers against rF1V **(** **Table 2** **)**. Based on the comparisons between the survival analysis and antibody response, a total IgG antibody titer of 3.58x10^5^, generated from 0.5 µg of rF1V, was sufficient to achieve 100% protection against CO92 challenge while only affording 50% protection against C12 challenge. An antibody titer of 1.27x10^6^ resulted in 80% survival in mice challenged with *Y. pestis* C12, one can therefore speculate that at an even higher antibody titer could achieve 100% protection against the capsule negative strain. A stronger Th1 mediated immune response (IgG2a/IgG1 Ratio) was induced at higher rF1V doses, 0.0158 (3.0 µg rF1V) > 0.0012 (0.5 and 1.0 µg F1V) **(** **Table 2** **)**. Furthermore, two lower vaccine doses, ranging from 0.5 – 3.0 µg, of rF1V (3.58x10^5^ - 1.27x10^6^) administered during this study produced a significantly greater antibody response than one high dose of 10 µg (1.66x10^5^) which was administered in the previous experiment.

**Table 2:** Antibody titers in BALB/c mice after double dose of rF1V vaccination.

Vaccine Treatment^a^	No. Survivors after Challenge		Antibody Titer^b^ ^,^ ^c^			Ratio IgG2a/IgG1
	*Y. pestis* CO92	*Y. pestis* C12	IgG	IgG1	IgG2a	
PBS	0/10	0/10	50 (1.00)	50 (1.00)	50 (1.00)	1.0000
0.5 μg rF1V	10/10	5/10	357,771 (1.19)	1,510,199 (1.14)	1,789 (1.19)	0.0012
1.0 μg rF1V	10/10	8/10	476,624 (1.37)	4,507,631 (1.12)	5,350 (1.38)	0.0012
3.0 μg rF1V	10/10	8/10	1,269,921 (1.10)	2,015,874 (1.19)	31,874 (1.14)	0.0158

a Two subcutaneous vaccinations.

b n was 4 for each group of mice.

c Antibody titer 28 days after vaccination. Reported as geometric mean (Geo Mean) with geometric standard error (GSE).

### Double Dose of rF1V With CpG Enhanced Both Humoral and Cellular Immune Response

Previous work has demonstrated that rF1V vaccine is able to induce an effective immune response against *Y. pestis* challenge by mainly inducing humoral immune response (26, 54, 55). High titers of anti-V antibodies are known to be critical for protection against virulent strains of F1-negative *Y. pestis* (11). Unfortunately, rF1V vaccination induces a very limited cell-mediated immune response which is why we postulate the poor and inconsistent protection (0-75%) of the vaccine in NHP (African green monkeys) against challenge with aerosolized *Y. pestis* (30). In order to achieve more robust protection against *Y. pestis* (especially against F1-negative strains) and elicit an anti-*Y. pestis* Th1 immune response, we tested rF1V in combination with various Toll-like receptor agonists. Due to the higher level of protection (8/10 mice survived, **Figure 3B**) afforded against *Y. pestis* C12 challenge with 1.0 and 3.0 µg rF1V doses, the following studies were designed using 0.5 µg of rF1V (5/10 mice survived, **Figure 3B**) in the presence of TLR agonists, in order to discern any potential immunological enhancement.

Previous studies demonstrated that our vaccine formulation is dependent on alhydrogel-rF1V complexing with immunomodulators in order to promote an effective immune response (19). Although, the amount of rF1V that would be required to saturate Alhydrogel is unknown, and it is plausible that unbound rF1V may be detrimental to producing an optimal immune response if it dissociates with Alhydrogel-rF1V complexes and competes for binding to antigen presenting cells. Therefore, we first sought to quantitate the amount of rF1V necessary to saturate Alhydrogel. Known quantities of the rF1V vaccine (5-100 µg) were added to PBS in the presence or absence of 100 µg of Alhydrogel. After incubation, the samples were centrifuged to pellet Alhydrogel-rF1V complexes, and the supernatant (unbound rF1V) was collected for mass spectrometry interrogation. We used the number of peptide spectral matches (PSMs) in each sample to provide relative quantitation of the amount of unbound F1 or V. The saturation point of 100 µg of Alhydrogel was only achieved with the addition of 50 to 100 µg of rF1V (**Figure S1**).

We performed our immunomodulatory studies with the addition of low (5 µg) or high (25 µg) dose of TLR agonists; Pam3CSK4 (TLR2), Poly(IC)LC (TLR3), MPLA (TLR4), Imiquimod (TLR7), R848 (TLR7/8) or CpG2006 (TLR9) to our rF1V formulation with which we proceeded to vaccinate BALB/c mice. We vaccinated BALB/c mice with two subcutaneous vaccine doses, 3 weeks apart. The groups were as follows: Control (PBS), 25 µg TLR agonist alone, 0.5 µg rF1V, 0.5 µg rF1V + 5 µg TLR agonist, or 0.5 µg rF1V + 25 µg TLR agonist. All vaccine preparations including the PBS group contained a reduced amount of *Alhydrogel (*250 µg/dose, down from 500 µg/dose used previously) based on the saturation data presented above (**Figure S1**). Both, the cytokine and antibody response were measured 28 days post boost vaccination in 5 mice from each group against rF1V. No adverse side-effects were noted post vaccination with the inclusion of the various TLR agonists.

**Figure 4:**
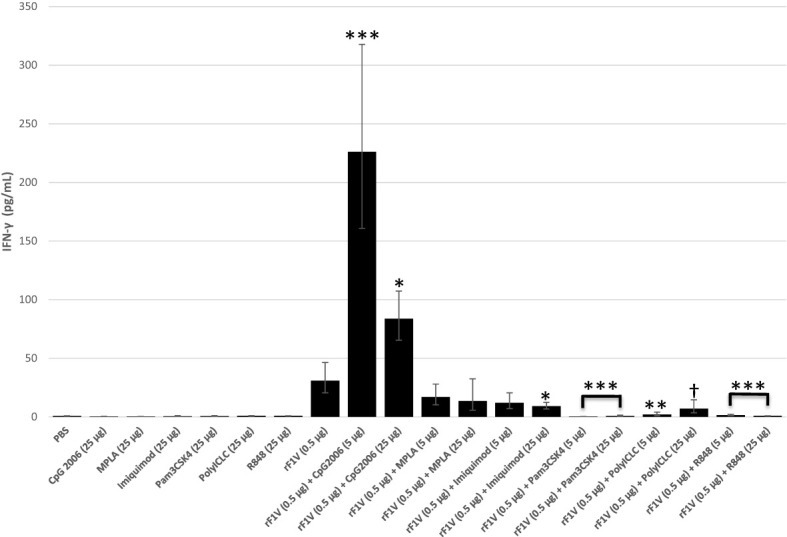
Production of IFN-γ by whole splenic lymphocytes from mice vaccinated with rF1V and TLR agonists. BALB/c mice were vaccinated twice, subcutaneously, 3 weeks apart with mock/PBS (n=15), rF1V (n=14), with and without 5 or 25 µg of TLR agonists (n=5). At 28 days post vaccination, mice were euthanized and spleens were harvested. Splenocytes (10^6^ cells) were re-stimulated in the presence of rF1V (25 µg/ml) for 48 hours. The levels of IFN-γ were measured by the Luminex bead-based suspension assay. Log transformed values were analyzed by two-way ANOVA (treatment x assay date) and results are reported as the geometric mean and geometric standard error. *p*-values are obtained from the main effect contrasts of the ANOVA model. No adjustment has been applied for multiple comparisons. ^†^p<0.05, *p<0.01, **p<0.001, ***p<0.0001 (Comparisons show significant differences *versus* the rF1V (0.5 µg) group).

There was enhanced IFN-γ production, in re-stimulated splenocytes from vaccinated mice, in the presence of either the higher (83.8 pg/mL, p<0.001) or more so, at the lower concentration (226.0 pg/mL, p<0.0001) dose of CpG2006 compared to rF1V only vaccinated group (30.9 pg/mL). There was no significant change in IFN- γ production in the presence of MPLA or low dose of Imiquimod, while there was a significant inhibition in IFN-γ levels in the presence of all other TLR agonists **(** **Figure 4** **)**.

Mice vaccinated with rF1V along with high dose (25 µg) of CpG2006, Imiquimod, R848, or with the low dose (5 µg) of Pam3CSK4 had demonstrably higher titers compared to mice vaccinated with rF1V alone **(** **Table 3** **)**. The highest IgG1 titers were produced in mice vaccinated with rF1V along with either, the low or the high CpG2006 doses, or with the high dose of Imiquimod. The IgG2a antibody titers increased in the presence of all TLR agonist although significance was not reached with the addition of lower dose of Pam3CSK4 or the lower or higher doses of R848. Although, the most pronounced increase in the IgG2a titer was only in the presence of CpG2006 (low dose 4.8x10^5^, p<0.0001; high dose 1.1x10^6^, p<0.0001) relative to rF1V only (1.9x10^4^) vaccinated group **(** **Table 3** **)**. Based on the IgG, IgG1 and IgG2a end point titer and the IgG2a/IgG1 ratio, the highest induction of a Th1 response was in the presence of CpG2006 with an increase of 13.5 and 30 fold in the presence of 5 µg and 25 µg CpG2006 relative to rF1V alone, respectively **(** **Table 3** **)**. Accordingly, CpG2006 was down selected for further vaccine formulations.

**Table 3:** Effect of Pam3CSK4 (TLR2), Poly(IC)LC (TLR3), MPLA (TLR4), Imiquimod (TLR7), R848 (TLR7/8) and CpG (TLR9) with rF1V vaccine on the F1V antibody response in BALB/c mice.

Vaccine Treatment^c^	Antibody Titer^a^ ^b^			Ratio IgG2a/IgG1
	Class	Isotype		
	IgG	IgG1	IgG2a	
PBS	50 (1)	50 (1)	50 (1)	1.000
CpG 2006 (25 pg)	61 (1)	50 (1)	63 (1)	1.260
MPLA (25 μg)	50 (1)	50 (1)	50 (1)	1.000
Imiquimod (25 μg)	50 (1)	50 (1)	50 (1)	1.000
Pam3CSK4 (25 μg)	50 (1)	50 (1)	52 (1.08)	1.040
Poly (IC)LC (25 μg)	50 (1)	50 (1)	50 (1)	1.000
R848 (25 μg)	50 (1)	50 (1)	50 (1)	1.000
rF1V (0.5 μg)	1,326,807 (1.33)	4,561,855 (1.34)	19,346 (1.35)	0.004
rF1V (0.5 μg) + CpG2006 (5 μg)	2,281,929 (1.12)	8,865,169 (1.22)*	479,587 (1.4)***	0.054
rF1V (0.5 μg) + CpG2006 (25 μg)	4,335,445 (1.12)**	9,332,232 (1.24)*	1,116,941 (1.24)***	0.120
rF1V (0.5 μg) + MPLA (5 μg)	2,224,093 (1.22)	6,047,733 (1.21)	82,080 (1.37)*	0.014
rF1V (0.5 μg) + MPLA (25 μg)	1,635,849 (1.21)	3,622,337 (1.33)	111,694 (1.39)**	0.031
rF1V (0.5 μg) + Imiquimod (5 μg)	2,425,147 (1.17)	4,215,755 (1.72)	116,148 (1.72)*	0.028
rF1V (0.5 μg) + Imiquimod (25 μg)	3,482,202 (1.27)^†^	16,025,318 (1.29)*	101,113 (1.26)***	0.006
rF1V (0.5 μg) + Pam3CSK4 (5 μg)	4,222,425 (1.17)**	7,650,820 (1.3)	45,948 (1.45)	0.006
rF1V (0.5 μg) + Pam3CSK4 (25 μg)	2,301,032 (1.16)	5,047,659 (1.19)	50,477 (1.31)*	0.010
rF1V (0.5 μg) + Poly (IC)LC (5 μg)	1,458,756 (1.5)	1,527,747 (1.28)*	105,728 (1.46)*	0.069
rF1V (0.5 μg) + Poly (IC)LC (25 μg)	1,918,763 (1.29)	3,045,846 (1.66)	132,578 (1.44)**	0.044
rF1V (0.5 μg) + R848 (5 μg)	1,098,561 (1.29)	1,921,799 (1.8)	87,746 (1.47)	0.046
rF1V (0.5 μg) + R848 (25 μg)	3,179,826 (1.14)*	11,596,475 (1.2)	139,288 (1.45)	0.012

a Values represent geometric mean (Geo Mean) with geometric standard error (GSE).

b All p-values are relative to the rF1V (0.5 μg) control group. ^†^p<0.05, *p<0.01, **p<0.001, ***p<0.0001.

c Values in parenthesis represent low (5 μg) and high (25 pg) toll-like receptor agonist amount per vaccine dose.

In an attempt to further characterize the anti-F1V antibody response, we independently examined the total IgG response against the two major antigens, the F1 and the V, that make up the rF1V vaccine. Furthermore, a previous study reported that the bulk of the IgG response generated by rF1V is against the V antigen of the rF1V vaccine (19). The anti-F1 antibody response with rF1V formulations containing TLR agonists was only marginally increased in the presence of either the low or the high dose or either CpG2006 or Imiquimod. Concordant with the previous study, the bulk of the antibody response was against the V-antigen. The most pronounced enhancement in anti-V IgG titer relative to rF1V only (4.2x10^5^) vaccinated mouse groups was in the presence of both the low and the high dose CpG2006 (low dose 1.4x10^6^, p<0.01; high dose 1.7x10^6^, p<0.0001) (19) **(** **Table 4** **)**. Although, other adjuvants also enhanced antibody titers against V, the enhancements were not as distinct or consistent for both adjuvant doses as they were for CpG2006 groups. Vaccination with rF1V in the presence of CpG promoted a strong IFN-γ response and a high anti-F1V IgG2a antibody titer, both of which are indicative of antigen specific Th1-like response recruitment (**Figure 4**, **Tables 3**, **4**).

**Table 4:** Effect of Pam3CSK4 (TLR2), Poly(IC)LC (TLR3), MPLA (TLR4), Imiquimod (TLR7), R848 (TLR7/8) and CpG (TLR9) with rF1V vaccine on F1 and V antibody response in BALB/c mice.

Vaccine Treatment^c^	IgG Titer^a^ ^b^	
	Anti-F1	Anti-V
PBS	50 (1)	50 (1)
CpG 2006 (25 μg)	50 (1)	50 (1)
MPLA (25 μg)	50 (1)	50 (1)
Imiquimod (25 μg)	50 (1)	50 (1)
Pam3CSK4 (25 μg)	57 (1.14)	50 (1)
Poly (IC)LC (25 μg)	57 (1.14)	50 (1)
R848 (25 μg)	50 (1)	50 (1)
rF1V (0.5 μg)	96,858 (1.32)	416,801 (1.34)
rF1V (0.5 μg) + CpG2006 (5 μg)	188,638 (1.27)	1,355,862 (1.41)*
rF1V (0.5 μg) + CpG2006 (25 μg)	552,067 (1.35)***	1,735,651 (1.26)***
rF1V (0.5 μg) + MPLA (5 μg)	167,692 (1.3)	1,333,309 (1.63)^†^
rF1V (0.5 μg) + MPLA (25 μg)	68,459 (1.21)	760,629 (1.38)
rF1V (0.5 μg) + Imiquimod (5 μg)	319,494 (1.51)^†^	459,479 (1.23)
rF1V (0.5 μg) + Imiquimod (25 μg)	242,515 (1.17)*	606,287 (1.17)
rF1V (0.5 μg) + Pam3CSK4 (5 μg)	48,045 (1.5)	418,918 (1.2)
rF1V (0.5 μg) + Pam3CSK4 (25 μg)	25,238 (1.51)^†^	801,266 (1.25)
rF1V (0.5 μg) + Poly (IC)LC (5 μg)	22,006 (2.08)	230,832 (1.67)
rF1V (0.5 μg) + Poly (IC)LC (25 μg)	126,191 (1.53)	504,766 (1.34)
rF1V (0.5 μg) + R848 (5 μg)	69,644 (1.45)	159,495 (1.31)^†^
rF1V (0.5 μg) + R848 (25 μg)	183,792 (1.53)	459,479 (1.14)

a Values represent geometric mean (Geo Mean) with geometric standard error (GSE).

b All p- values are relative to the rF1V (0.5 Mg) control group. ^†^p <0.05, *p <0.01, ***p <0.0001.

c Values in parenthesis represent low (5 μg) and high (25 μg) toll-like receptor agonist amount per vaccine dose.

### Addition of CpG to rF1V Improves Protection Against *Y. pestis* C12 Challenge

**Figure 5:**
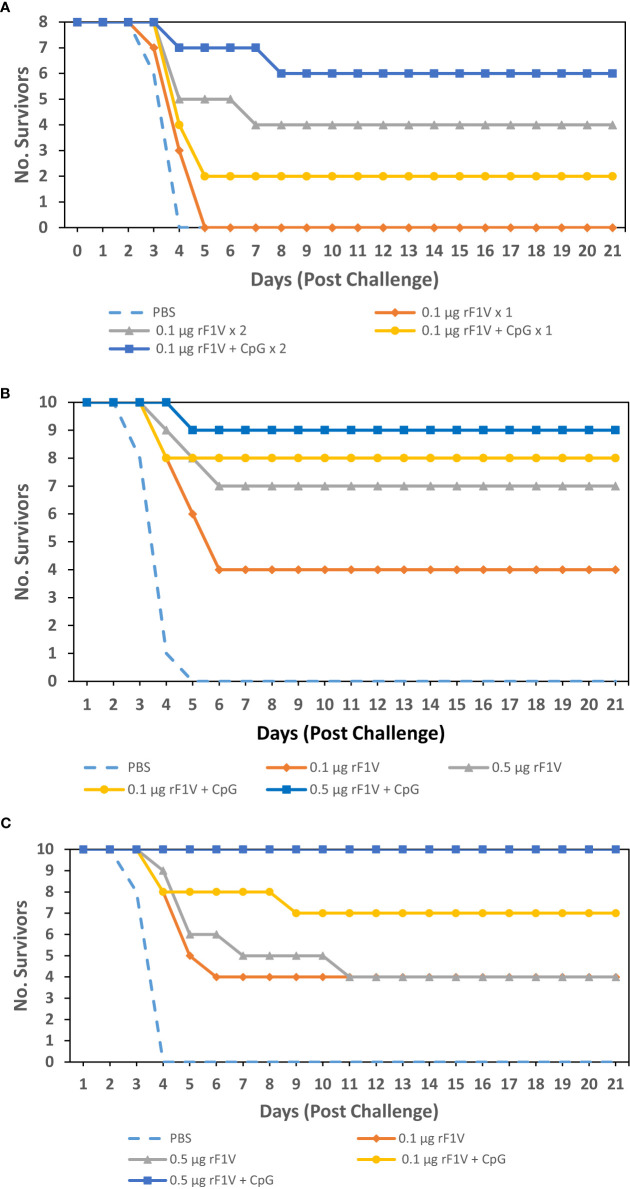
Adjuvanation of rF1V with CpG improves protection against *Y. pestis* C12 aerosol challenge. **(A)** BALB/c mice were vaccinated with either one subcutaneous dose (x 1) or 2 subcutaneous doses (x 2) that were 3 weeks apart, with mock/PBS, 0.1 µg or 0.5 µg of rF1V with or without 5 µg CpG (n=8). At 29 days post vaccination, BALB/c mice were exposed to 4.81x10^5^ aerosolized CFU (approximately 6 LD_50_ equivalents) of *Y. pestis* C12. **(B)** BALB/c mice were vaccinated with 2 subcutaneous doses that were 3 weeks apart (n=10). At 29 days post vaccination, BALB/c mice were exposed to 6.51x10^5^ aerosolized CFU (approximately 8 LD_50_ equivalents) of *Y. pestis* C12. **(C)** BALB/c mice were vaccinated with 2 subcutaneous doses that were 3 weeks apart (n=10). At 43 days post vaccination, BALB/c mice were exposed to 2.25 x10^5^ aerosolized CFU (approximately 3 LD_50_ equivalents) of *Y. pestis* C12. Survival was monitored for 21 days post challenge.

Lastly, in order to determine if the immune enhancement observed with the inclusion of CpG2006 translates to increased protection against *Y. pestis* C12, BALB/c mice were vaccinated with two subcutaneous vaccine doses of 0.1 µg rF1V +/- 5 µg CpG2006, 3 weeks apart. In addition, to demonstrate the enhanced efficacy of double dose over single dose vaccine regiments mice were vaccinated with a single dose of 0.1 µg rF1V +/- 5 µg CpG2006. The vaccinated mice were challenged with aerosolized bacteria 29 days post vaccination with *Y. pestis* C12 (Inhaled dose approximately 6 LD_50_s [4.81x10^5^ CFU], **Figure 5A**). Mice were monitored for 21 days post challenge. Four out of 8 mice vaccinated with a double dose (0.1 µg rF1V x 2) vaccine survived relative to 0/8 survivors after single dose (0.1 µg rF1V x 1) vaccination. Although, this difference in survival rate did not reach statistical significance (p=0.0769 *vs* 0.1 µg rF1V x 1) likely due to group size (N=8 in this iteration), the median time to death or euthanasia of the double dose proved to be statistically significant relative to both the single dose and control (PBS) vaccinated mice (p=0.0187 *vs* 0.1 µg rF1V x 1, p=0.0061 *vs* PBS). Addition of CpG to the single dose of rF1V enhanced survival (0.1 µg rF1V + CpG [2/8 survived] *vs* 0.1 µg rF1V x1 [0/8 survived]) but this did not reach significance. Double dose of rF1V with CpG (0.1 µg rF1V + 5 µg CpG x 2) conferred the highest level of protection (6/8 survived) reaching significance relative to single dose of rF1V (p=0.007 *vs* 0.1 µg rF1V x 1) furthermore, the median time to death or euthanasia was also enhanced attaining significance against the single doses of rF1V +/- CpG (p=0.0008 *vs* 0.1 µg rF1V x 1, p=0.0377 *vs* 0.1 µg rF1V + CpG x 1).

The enhancement in the number of rF1V specific IFN-γ secreting cells was also evident with the addition of a second vaccine dose (p=0.0146 0.1 µg rF1V x 1 *vs* 0.1 µg rF1V x 2), as determined by an *ex vivo* ELISpot assay (**Figure S2**). The IFN-γ response was further enhanced with the addition of CpG to the vaccine formulation (0.1 µg rF1V + CpG x 2 *vs* 0.1 µg rF1V x 1 [p<0.0035]. These results are also in concordance with the increased IFN-γ secretion levels in the presence of CpG, described previously (**Figure 4**).

**Figure 6:**
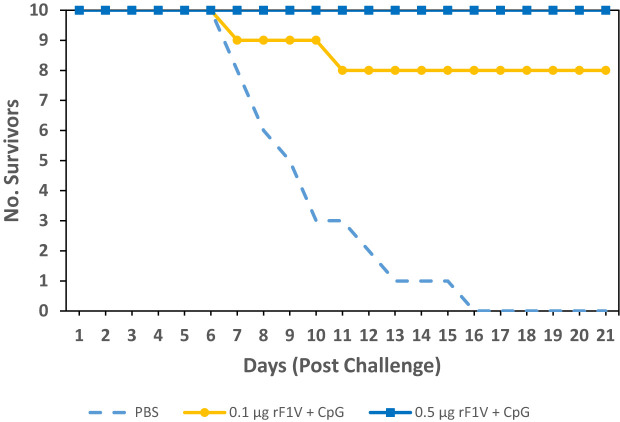
Adjuvanation of rF1V with CpG improves protection against *Y. pestis* C12 subcutaneous challenge. BALB/c mice were vaccinated with two subcutaneous doses, 3 weeks apart with mock/PBS, 0.1 µg or 0.5 µg of rF1V with 5 µg CpG (n=10). At 29 days post vaccination, BALB/c mice were subcutaneous challenged with 3.6x10^3^ CFU (approximately 400 LD_50_ equivalents) of *Y. pestis* C12. Survival was monitored for 21 days post challenge.

In an effort to further enhance the level of protection the double dose of rF1V vaccine was increased from 0.1 µg to 0.5 µg. BALB/c mice were vaccinated with two subcutaneous vaccine doses, 3 weeks apart, and challenged with *Y. pestis* C12. The groups, adjuvanted with Alhydrogel, were as follows: Control (PBS), 0.1 µg rF1V +/- 5 µg CpG2006, and 0.5 µg rF1V +/- 5 µg CpG2006. The vaccinated mice were challenged with aerosolized bacteria 29 days post vaccination with *Y. pestis* C12 (Inhaled dose approximately 8 LD_50_s [6.51x10^5^ CFU], **Figure 5B**). Mice were monitored for 21 days post challenge. Statistical significance was not observed in mice vaccinated with 0.1 µg rF1V (4/10 survived, p=0.0867 *vs* PBS) while statistical significance was reached in mice vaccinated with 0.5 µg rF1V (7/10 survived, p=0.0031 *vs* PBS). Addition of CpG to rF1V improved the survival of vaccinated mice post *Y. pestis* C12 exposure, with 8/10 (p=0.0007 *vs* PBS) of 0.1 µg rF1V + CpG vaccinated mice and 9/10 (p=0.0001 *vs* PBS) of 0.5 µg rF1V + CpG vaccinated mice surviving the challenge. Furthermore, mice vaccinated with 0.1 µg rF1V + CpG and 0.5 µg rF1V + CpG survived a high subcutaneous challenge dose (dose approximately 400 LD50s [3,620 CFU]) with *Y. pestis* C12 on day 29 post-vaccination resulting in 8/10 (p=0.0007 *vs* PBS) and 10/10 (p<0.0001 *vs* PBS) mice surviving, respectively (**Figure 6**).

An additional set of vaccinated mice were challenged with aerosolized bacteria 43 days post vaccination with a lower dose of *Y. pestis* C12 (Inhaled dose = 2.25x10^5^ CFU or approximately 3 LD_50_ equivalents, **Figure 5C**). Mice were monitored for 21 days post challenge. Statistical significance was not observed in mice vaccinated with 0.1 µg rF1V or 0.5 µg rF1V (4/10 survived, p= 0.0867 *vs* PBS). Once against, addition of CpG to rF1V improved the survival of vaccinated mice post *Y. pestis* C12 exposure even with a greater time between vaccination and bacterial challenge, with 7/10 (p=0.0031 *vs* PBS) of 0.1 µg rF1V + CpG vaccinated mice surviving, while mice vaccinated with 0.5 µg rF1V + CpG were fully protected against challenge (10/10 survived; p<0.0001 *vs* PBS). Therefore, addition of CpG to rF1V in the presence of alhydrogel enhances the level of protection conferred by rF1V vaccination.

## Discussion

Vaccination with the rF1V subunit vaccine candidate is a safe and promising strategy resulting in protection against *Yersinia pestis* infection. However, unlike the historical rF1V or rV bench-scale preparations generated at the USAMRIID that demonstrated equivalent protection of mice challenged with *Y. pestis* CO92 or *Y. pestis* C12 the most recent cGMP rF1V formulation routinely protects less mice challenged with *Y. pestis* C12 compared to those challenged with *Y. pestis* CO92 (11, 17). In our hands, the rF1V appears to generate a weak cell mediated immune response and this may explain the limited protection against F1-negative strains of *Y. pestis*. Furthermore, Elvin et at. demonstrated poor protection in vaccinated mice that have a targeted gene deletion in the signal transducer and activator of transcription 4 (Stat 4-/-) post challenge, while the vaccine conferred protection in State 6-/- mice. The Stat 4-/- mice are not able to properly utilize IL-12 and IFN-y and are predisposed to a Th2-like immune response profile, while the Stat 6-/- mice are not able to use IL-4 and IL-13 and have reduced levels of IgG1 and hence have a biased Th1-like response (56). Our initial assessment identified rF1V vaccinated BALB/c mice to be more susceptible to *Y. pestis* CO92 relative to rF1V vaccinated outbred CD-1 mice **(** **Figure 1A** **)**. This enhanced level of protection in CD-1 mice relative to BALB/c mice may have been partially attributed to the overall higher antibody titers against rF1V **(** **Table 1** **)**. The predominant antibody response against rF1V was IgG1 in both strains of mice. BALB/c mice generated slightly higher IgG2a antibody titers as compared to CD-1 mice. The greater susceptibility of BALB/c mice to *Y. pestis* made this mouse strain more pertinent for continuation of vaccine efficacy assessments since any future vaccine modifications that confer greater protection to the more susceptible animal model should translate to similar or greater protection in the less susceptible animal model.

Since prior work alluded to rF1V vaccination providing animals with reduced levels of protection against the F1-negative *Y. pestis* C12 stain we proceeded to compare the level of protection conferred by rF1V vaccination against *Y. pestis* CO92 and *Y. pestis* C12 challenge. The level of protection in mice vaccinated with a single dose of rF1V was reduced when challenged with *Y. pestis* C12 relative to mice challenged with *Y. pestis* CO92 **(** **Figure 2** **)**. By modifying the vaccine schedule with the addition of a boost, the dose of the rF1V vaccination could be reduced, thus providing the opportunity for dose sparing. Antibody titers were substantially increased after the prime-boost regiment relative to the prime only vaccine schedule **(** **Tables 1**, **2** **)**. However, protection was still less when the vaccinated mice were challenged with aerosolized *Y. pestis* C12 relative to mice challenged with *Y. pestis* CO92 **(** **Figure 3** **)**.

In an effort to circumvent the limitations of rF1V vaccine in protection against *Y. pestis* C12 we tested an array of Toll-like receptor agonists with the goal of stimulating a more robust Th1-like immune response and enhance protection against F1-negative *Y. pestis* strains. TLR agonists are known to enhance the efficacy of vaccines by helping to activate the immune cells by mimicking pathogen associated molecular patters (PAMPs). Of the six immunomodulators tested the TLR 9 agonist, CpG2006, produced the strongest IFN-γ response at both concentrations tested, although the response was greatest at the lower concertation **(** **Figure 4** **)**. High levels of IFN-γ are indicative of Th1-like response stimulation. Furthermore, the induction of a stronger IFN-γ response in the presence of lower concentration of CpG2006 (5 µg) may be partially attributed to the oversaturation of Alhydrogel at the higher concentration of CpG2006 (25µg) resulting in inadvertent competition between CpG2006 and rF1V for the binding to Alhydrogel and hence reduction in rF1V interaction with Alhydrogel. Our preliminary results indicate that 100 µg/ml of Alhydrogel is saturated with CpG2006 in the range between 10 to 25 ug (**Table S3**).

Of note, currently the CpG ODNs are grouped into three classes (CpG-A, CpG-B and CpG-C) that are based on varying structural characteristics and immunostimulatory effects (57). CpG-A promotes NK cell activation, high IFN-α production from plasmacytoid dendritic cells but are poor stimulators of both B cells and the NF-κB pathway activation (58–60). In contrast, the CpG-B class of molecules, of which CpG2006 belongs to, induces strong stimulation of B-cells and TLR9-dependent NF-κB signaling but poor induction of IFN-α secretion (61–64). CpG-C is an amalgam of structural features of both CpG-A and CpG-B, thereby able to induce both a strong IFN-α production and B-cell activation (65, 66).

All TLR agonists tested, with the exception of the lower dose of Poly(IC)LC (TLR3) and R848 (TLR 7/8), to some degree promoted a stronger anti-F1V IgG antibody response in mice over that of mice vaccinated with rF1V alone. The anti-F1V IgG1 response was predominantly enhanced with the inclusion of almost all TLR agonists but the enhancement in titers was most consistent with the addition of CpG2006 at both concentrations tested. The most prominent difference that was observed in the presence of TLR agonist was in the increase of IgG2a titers in comparison to rF1V only vaccinated mice. Furthermore, addition of CpG2006 to rF1V significantly increased the IgG2a antibody titers. The increase of IgG2a isotype production further indicates the stimulation of Th1-like immune response. Adjuvanticity of CpG promoted a more Th1-like response based on the increase of the IgG2a/IgG1 ratio, which is a reflection of the Th1/Th2 immune polarization (67). Since rF1V is a chimera of F1 and V, we determined the antibody response to each of the individual proteins. The addition of TLR agonists specifically CpG (TLR9) and Imiquimod (TLR7) increased anti-F1 antibody titers while the addition of CpG greatly increased anti-V titers. Although, addition of MPLA to rF1V did not enhance IFN-γ response, it did increase the anti-V antibody titers relative to rF1V only treatment. It is possible that inclusion of both CpG2006 and MPLA to the rF1V vaccine formulation may additively or synergistically increase protection against a F1-negative *Y. pestis* strains.

The ultimate goal of this study was to assess and improve the level of protection against challenge with aerosolized F1-negative *Y. pestis* C12 strain in rF1V vaccinated mice. With the inclusion of CpG2006 in the rF1V vaccine formulation, there was an enhancement in the level of protection against *Y. pestis* C12 challenge (**Figure 5**). The increase in the level of protection was associated with both the increase in the dose of rF1V and the presence of CpG2006. Furthermore, inclusion of CpG2006 in the rF1V formulation also conferred robust protection in subcutaneous *Y. pestis* C12 challenge, in the BALB/c mouse bubonic plague model (**Figure 6**).

Future work will be directed at refining rF1V immune response and enhancing protection of vaccinees may investigate combinations of adjuvants, such as a mixture of CpG with MPLA, adjusting the vaccine regiment to include additional boost vaccinations, supplementing the F1 and V proteins with additional protective *Y. pestis* antigens or potentially pairing the subunit rF1V with promising live attenuated vaccines. Additional effort should focus on elucidating the induction of Th17 response by the rF1V vaccine, specifically at the lung mucosal sites, since the production of IL-17 and IL-22 has been shown to correlate with vaccine-induced protection against aerosolized *Y. pestis* (68–71). The magnitude of Th17 response may also enhance secretory IgA titers in the lungs, which may contribute to vaccine efficacy (72–74).

Importantly, there are examples of differences in immune responses generated by vaccines associated with sex (75–77). Bowen et al. demonstrated statistically significant sex-biased survival differences observed in rF1V vaccinated BALB/c and C57BL/6 after intranasal instillation with *Y. pestis* (78). These authors demonstrated that vaccinated female mice were significantly more likely to survive challenge with virulent *Y. pestis* when compared to vaccinated male mice. While the authors could not elucidate the reason for these differences, these differences must be explored later in more advanced studies. While we only examined female mice in this current study, male mice will be examined during advanced development of novel vaccine formulations such as those described here.

In conclusion, the addition of CpG2006, enhanced both IFN-γ and IgG2a production in rF1V vaccinated mice. The observed dual humoral and CMI enhancement makes CpG2006 a candidate for inclusion with rF1V vaccination.

## Data Availability Statement

The raw data supporting the conclusions of this article will be made available by the authors, without undue reservation.

## Ethics Statement

Research was conducted under an IACUC approved protocol in compliance with the Animal Welfare Act and other federal statutes and regulations related to animals and experiments involving animals. The facility where this research was conducted is accredited by the Association for Assessment and Accreditation of Laboratory Animal Care International and adheres to principles stated in the Guide for the Care and Use of Laboratory Animals, National Research Council, 2011.

## Author Contributions

Conceptualization: CKC, SSB, KA, PLW; methodology: CKC, SSB, KA; formal analysis: CKC, SSB, KA, DPF; investigation: CKC, KA, JAB, SSB, MH, ZS, IV, NOR, CPK, JLS, RRA, JLD, LHC; resources: PLW, CKC; data curation: CKC, SSB; writing—original draft preparation: CKC and SSB; writing—review and editing: CKC, SSB, JAB, MDW, LHC, ; supervision: CKC; project administration: CKC; funding acquisition: PLW and CKC. All authors have read and agreed to the published version of the article.

## Author Disclaimer

Opinions, interpretations, conclusions, and recommendations are those of the authors and are not necessarily endorsed by the U.S. Army.

## Conflict of Interest

The authors declare that the research was conducted in the absence of any commercial or financial relationships that could be construed as a potential conflict of interest.

## Publisher’s Note

All claims expressed in this article are solely those of the authors and do not necessarily represent those of their affiliated organizations, or those of the publisher, the editors and the reviewers. Any product that may be evaluated in this article, or claim that may be made by its manufacturer, is not guaranteed or endorsed by the publisher.
