# Supplementary material for: Impact of Toll-Like Receptor-Specific Agonists on the Host Immune Response to the Yersinia pestis Plague rF1V Vaccine
Source: Front Immunol. 2021 Aug 27;12:726416. doi: 10.3389/fimmu.2021.726416 (PMC8430260; doi:10.3389/fimmu.2021.726416)
Supplement: Supplementary file 1 [file Presentation_1.pdf]

Table S1:

Toll-like receptor (TLR) Agonist			
**Name**	**Synthetic Ligand**	**Receptor**	**Physiological Ligand**
Pam3CSK4	triacylated lipopeptide	TLR 2	cell-wall components, such as lipoproteins
Poly(IC)LC	double-stranded polyriboinosinic-polyribocytidylic acid	TLR 3	double-stranded RNA
MPLA	Monophosphoryl Lipid A	TLR 4	lipopolysaccharide
Imiquimod	imidazoquinoline amine analog to guanosine	TLR 7	ssRNA and oligoribonucleotides
Resiquimod (R848)	imidazoquinoline amine analog to guanosine	TLR 7/8[a]	ssRNA and oligoribonucleotides
CpG2006	class B CpG oligonucleotide	TLR 9	unmethylated CpG dinucleotides

[a.] In mice, TLR8 appears to recognize different ligands relative to the human counterpart. In humans, Resiquimod activates both TLR7 and TLR8 but it only activates TLR7 in mice.

Table S2: The LD_{50} of *Y. pestis* CO92 and C12 in BALB/c and CD1 mice exposed via whole body aerosol or subcutaneous challenge.

**Mouse strain**	**Route of exposure**	**Bacterial strain**	**LD_{50} (CFU)[a]**	**Confidence intervals (CFU)[a]**
BALB/c	Aerosol	*Y. pestis* CO92	$6.8x10^4$ [b]	ND[b]
BALB/c	Aerosol	*Y.pestis* C12	$7.7x10^4$	ND [c]
BALB/c	Subcutaneous	*Y. pestis* CO92	1.6	0.62 - 4.1
BALB/c	Subcutaneous	*Y.pestis* C12	9.01	2.2 - 36.6
CD-1	Aerosol	*Y. pestis* CO92	$3.4x10^4$	$2.4x10^4$- $4.8x10^4$
CD-1	Aerosol	*Y.pestis* C12	$4.1x10^4$	$3.2x10^4$- $5.2x10^4$
CD-1	Subcutaneous	*Y. pestis* CO92	52.4	7.1 - $3.9x10^2$
CD-1	Subcutaneous	*Y. pestis* C12	$9.4x10^2$	$8.7x10^1$- $1.0x10^4$

[a] colony-forming unit

[b] Source: Heine, H.S., et al., Comparison of 2 antibiotics that inhibit protein synthesis for the treatment of infection with Yersinia pestis delivered by aerosol in a mouse model of pneumonic plague. The Journal of infectious diseases, 2007. 196(5): p. 782-787.

[c] The confidence interval was unbounded.

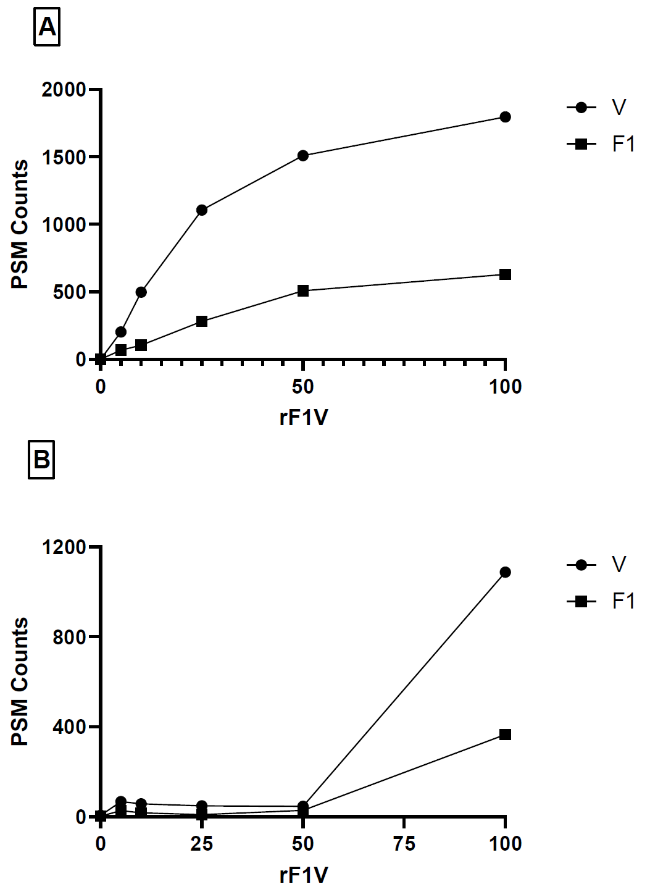

Figure S1. LC-MS/MS quantitation of V and F1 antigens using PSM and relative quantitation. (A) In the absence of alhydrogel and (B) in the presence of alhydrogel.

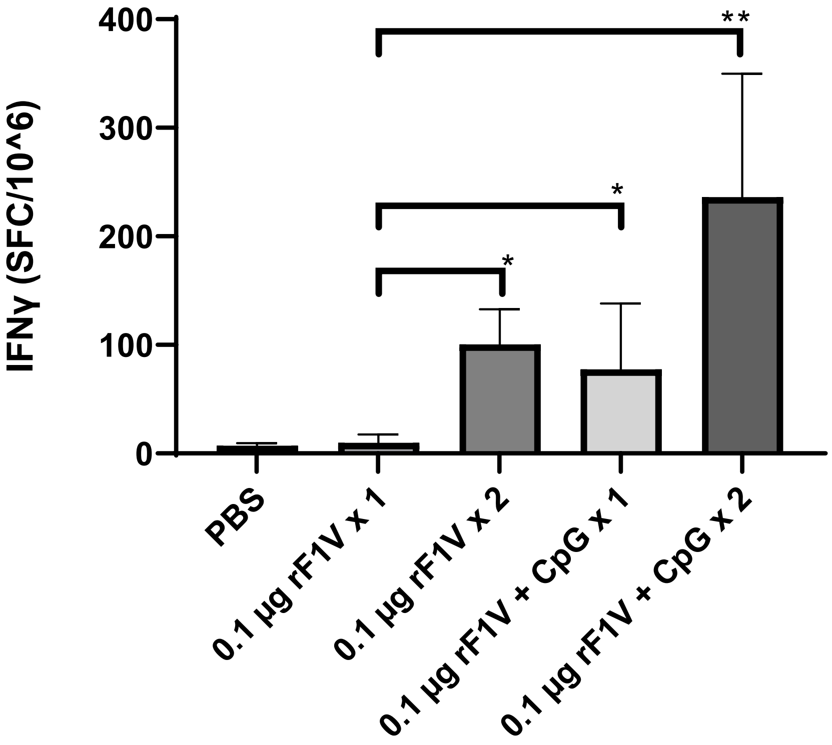

Figure S2. Double dose of rF1V with CpG enhanced recall response. Enumeration of IFN-γ response in secreting spleen cells stimulated by rF1V in the ELISpot assay. BALB/c mice (n=5 for all groups with the exception of 0.1 µg rF1V x 1 group which was n=4) were vaccinated, with either one (x 1) or two (x 2) doses that were 3 weeks apart, as described previously. Splenocytes were stimulated in the presence rF1V (25 µg/ml) for 24 hours at 37°C. The number of discreet IFN-γ secreting cells on the membrane were measured as spot-forming cells (SFC) per 10^6 cells. Data are presented as geometric mean of number of spots per well. p-values reflect the result of post-hoc comparisons under a repeated measures ANOVA model. Comparison between groups is made by Welch's t-test. *$p<0.05$, **$p<0.005$ (Comparisons show significant differences versus the 0.1 µg rF1V x 1 group)

Table S3: CpG saturation of 100 µg Alhydrogel.

Sample no.	Amount DPBS	Amount CpG2006 or MPLA	Amount Alhydrogel	Concentation (µg/ml)[a]
Order of addition:	(1)	(2)	(3)	
1	1000 µl	0	0	**-0.1**
2	980 µl	5.0 µg (20 µl)	0	**3.5**
3	960 µl	10 µg (40 µl)	0	**7.3**
4	900 µl	25 µg (100 µl)	0	**21.2**
5	800 µl	50 µg (200 µl)	0	**49.9**
6	600 µl	100 µg (400 µl)	0	**98.3**
7	950 µl	0	100 µg (50 µl)	**-0.2**
8	930 µl	5.0 µg (20 µl)	100 µg (50 µl)	**-0.1**
9	910 µl	10 µg (40 µl)	100 µg (50 µl)	**0**
10	850 µl	25 µg (100 µl)	100 µg (50 µl)	**7.9**
11	750 µl	50 µg (200 µl)	100 µg (50 µl)	**32.1**
12	550 µl	100 µg (400 µl)	100 µg (50 µl)	**82.9**

[a]Known quantities of the CpG2006 (5-100 µg) were added to 100 µg of Alhydrogel in a total volume of 1ml DPBS and incubated for 60 min at 4°C under rotational mixing. After the incubation the samples were centrifuged at 10,000 rpm (9,300 xg) for 10 min at 4°C and the supernatant was removed and placed in low protein binding tubes on ice. Concentration of free CpG2006 was determined by NanoDrop One UV-spectrum.
